# Programmed Cell Death Protein 1 Axis Inhibition in Viral Infections: Clinical Data and Therapeutic Opportunities

**DOI:** 10.3390/vaccines10101673

**Published:** 2022-10-07

**Authors:** Konstantinos Tsiakos, Niki Gavrielatou, Ioannis A. Vathiotis, Loukas Chatzis, Stamatios Chatzis, Garyfallia Poulakou, Elias Kotteas, Nikolaos K. Syrigos

**Affiliations:** 1 3rd Department of Internal Medicine, Medical School, National and Kapodistrian University of Athens, 157 72 Athens, Greece; 2 Department of Pathology, School of Medicine, Yale University, New Haven, CT 06520, USA; 3 Pathophysiology Department, Athens School of Medicine, National and Kapodistrian University of Athens, 157 72 Athens, Greece; 4 Department of Internal Medicine, Medical School, National and Kapodistrian University of Athens, “Hippokration” Hospital, 115 27 Athens, Greece; 5 Dana-Farber Brigham Cancer Center, Boston, MA 02215, USA; 6 Harvard Medical School, Boston, MA 02215, USA

**Keywords:** immunotherapy, immune checkpoint molecules, immune checkpoint inhibitors, ICIs, viral infections, cancer

## Abstract

A vital function of the immune system is the modulation of an evolving immune response. It is responsible for guarding against a wide variety of pathogens as well as the establishment of memory responses to some future hostile encounters. Simultaneously, it maintains self-tolerance and minimizes collateral tissue damage at sites of inflammation. In recent years, the regulation of T-cell responses to foreign or self-protein antigens and maintenance of balance between T-cell subsets have been linked to a distinct class of cell surface and extracellular components, the immune checkpoint molecules. The fact that both cancer and viral infections exploit similar, if not the same, immune checkpoint molecules to escape the host immune response highlights the need to study the impact of immune checkpoint blockade on viral infections. More importantly, the process through which immune checkpoint blockade completely changed the way we approach cancer could be the key to decipher the potential role of immunotherapy in the therapeutic algorithm of viral infections. This review focuses on the effect of programmed cell death protein 1/programmed death-ligand 1 blockade on the outcome of viral infections in cancer patients as well as the potential benefit from the incorporation of immune checkpoint inhibitors (ICIs) in treatment of viral infections.

## 1. Introduction

A vital function of the immune system is the modulation of an evolving immune response. Its dysregulation may lead to excessive tissue damage and induce autoimmunity. In its totality, the immune system is responsible for guarding against a wide variety of pathogens as well as for the establishment of memory responses to some future hostile encounters. Simultaneously, it maintains self-tolerance and minimizes collateral tissue damage at sites of inflammation [1,2,3]. These antagonistic functions are orchestrated by different subsets of T-cells, which roughly include the effector T-cells (Teff) and the regulatory T-cells (Tregs), with the former mediating the adaptive immunity and the latter being in charge of attenuating the immune response. Unquestionably, the dynamic ratios of T-cell subsets, including the Teff/Treg ratio, are important for maintaining effective immunity as well as self-tolerance [2].

In recent years, regulation of T-cell responses to foreign or self-protein antigens and the maintenance of balance between T-cell subsets have been linked to a distinct class of cell-surface and extracellular components, named “immune checkpoint molecules”. The term refers to a group of proteins that are mainly expressed on immune cells and are capable of regulating the immune response [3]. Immune checkpoints have been shown to activate downstream signaling pathways which affect immune cell survival, differentiation, and metabolism as well as cell-to-cell interactions [4,5,6,7,8]. Moreover, signaling through these molecules can force immune effector cells into “exhaustion”. T-cell exhaustion represents a transcriptionally distinct state, defined by sustained expression of inhibitory receptors and poor effector cell function [9].

**Figure 1:**
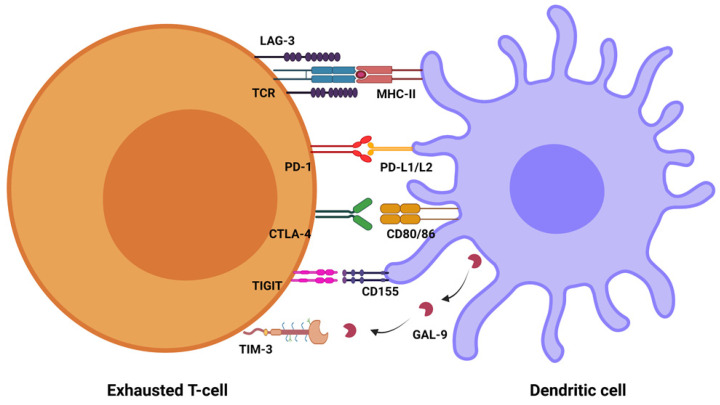
Immune checkpoint expression by exhausted T-cells in response to chronic viral infection. PD-1: programmed cell death protein 1, PD-L1/L2: programmed death-ligand 1/2, CTLA-4: cytotoxic T lymphocyte antigen 4, LAG-3: lymphocyte activated gene-3, GAL-9: galectin-9, TIM-3: T-cell immunoglobulin mucin-3, TIGIT: T-cell immunoglobulin and immunoreceptor tyrosine-based inhibitory motif, TCR: T-cell receptor, MHC-II: major histocompatibility complex.

Immune checkpoint molecules have been also found to be expressed across different types of solid tumors and are thought to be key players in tumor evolution by mediating the induction of malignant traits, including epithelial-to-mesenchymal transition (EMT) [10,11,12,13], relentless proliferation [14,15,16], evasion of cell death [17,18,19], metabolic reprogramming [20,21,22], induction of angiogenesis [23], and the processes of dissemination and metastasis [24,25]. Importantly, it has become evident that pathogens exploit immune checkpoint molecules to escape a host’s immune system as well (Figure 1). Following the work by Cai et al., this review focuses on the programmed cell death protein 1 (PD-1)/programmed death-ligand 1 (PD-L1) checkpoint and summarizes existing clinical evidence on the use of PD-(L)1 inhibitors in patients with cancer and select viral infections [26].

## 2. PD-1/PD-L1

Mainly expressed on CD4+ and CD8+ T-cells, B-cells, natural killer (NK) cells, monocytes, macrophages, and dendritic cells (DCs), programmed cell death protein 1 (PD-1) was first described in 1992 as a mediator of apoptosis [27,28,29]. Its expression can be induced by either T- or B-cell receptor signaling in the setting of antigen presentation or by cytokines (i.e., IL-2, IL-7, IL-15, and IL-21) in an antigen-independent manner [30]. Upon interaction with its ligands, PD-1 recruits tyrosine phosphatase Src homology 2 domain-containing phosphatases ½ (SHP1/2) that directly suppress the activation of the PI3K/Akt pathway and attenuate downstream TCR signaling, ultimately leading to decreased T-cell proliferation and survival, as well as reduced effector cell function and diminished IL-2 production. PD-1 ligands PD-L1 and PD-L2 exhibit distinct expression patterns. PD-L1 can be found both on tumor cells and immune cells, including T- and B-cells, macrophages, DCs, and bone-marrow-derived mast cells [31]. In contrast, PD-L2 expression is mostly limited to activated DCs, macrophages, and bone-marrow-derived mast cells [32]. The expression of PD-1 ligands is governed by the interferon signaling pathways, with PD-L1 being principally regulated by the interferon gamma-JAK-STAT-IRF1 axis and PD-L2 responding equally to interferon beta and gamma stimulation [33,34]. 

The PD-1/PD-L1 pathway relays an inhibitory signal that controls both central and peripheral immune tolerance. This led to the hypothesis that blockade of the PD-1/PD-L1 immune checkpoint limits tumor capacity to evade host immunity and produce deep and sustained antitumor responses [35]. The use of the first anti-PD-1 monoclonal antibodies (nivolumab and pembrolizumab) in cancer therapeutics was approved by the Food and Drug Administration (FDA) in 2014 [36]. Since then, both the number of agents and the list of indications have been expanding.

## 3. COVID-19

In recent years, the ongoing COVID-19 pandemic has caused cataclysmic shifts in everyday life, severely damaging the world economy and posing unprecedented challenges to the healthcare system. COVID-19 is currently considered the third leading cause of death in the US after diseases of the heart and cancer. Malignancies and COVID-19 are interconnected, with cancer patients not only suffering the collateral effects of the outbreak (disruption of prevention facilities and delayed diagnosis, hindered access to oncologic care, reduced access to medicines) but also confronted by an increased risk for severe COVID-19 [37,38]. Recently, it was shown that cancer patients with COVID have higher ICU admission rates, are more often in need of mechanical ventilation, and have higher mortality [39]. Those outcomes can be attributed to the underlying neoplastic process and poor performance status of the patients, the side effects of specific anti-tumor agents, or the combination of the two [40]. Consequently, it is critical to decipher the interactions between COVID-19 infection and malignancy to successfully guide clinical decisions towards the best outcome for both pathologic conditions [41]. Large cohort studies have shown that recent cytotoxic chemotherapy leads to more COVID-19-related poor outcomes [42,43]. However, data on checkpoint inhibitors have been conflicting. As the case remains compelling, we examine the link between the recently introduced immune checkpoint inhibitors (ICIs) and COVID-19.

Patients with severe or critical COVID-19 exhibit an abundance of immunological manifestations. Lymphopenia, T-cell exhaustion, and an upregulation of proinflammatory cytokines all lead to an aberrant immune response [44,45]. In the early stages of infection, even though the number of, mainly CD8+ but also CD4+, circulating T-cells is reduced, T-cell activity is overall increased. In severe infections, however, CD8+ T-cells shift to an exhausted phenotype impairing normal host defenses [46,47,48]. Overexpression of several immune checkpoint molecules, an indicator of T-cell exhaustion, has been implicated in the severity of COVID 19 infection and the associated lymphopenia [46,49,50]. Upregulation of inhibitory receptors, such as PD-1, has been observed in both nasopharyngeal swabs and lung tissue from autopsies of COVID-19 patients, with the degree of expression directly linked to the viral load [51]. Since ICIs enhance the immune response by interrupting the delivery of inhibitory signals and reinvigorating exhausted T-cells, they might represent promising candidates for incorporation in the COVID-19 treatment armamentarium [52]. However, the use of ICIs can result in drug-induced autoimmune pneumonitis, with an overall incidence of around 5%, and could hypothetically perpetuate or even trigger a cytokine storm, hindering its use as a COVID-19 treatment option [53]. In 2020, the race to uncover effective treatments for the COVID-19 outburst turned scientific focus in restoring host immune competence and resulted in the investigation into repurposing ICIs in several phase II clinical trials (three for nivolumab, one for pembrolizumab, one for camrelizumab) (Table 1) [54,55,56]. The results of the Copernico trial were recently published, showing that the use of pembrolizumab in combination with tocilizumab (an IL6 receptor antibody) in COVID-19 hospitalized, non-mechanically ventilated patients with severe pneumonia reduced the duration of hospital stay compared to the Standard of Care. However, the robustness of the study’s conclusion was limited by the low patient enrolment (seven patients in the treatment group). The results of the remaining studies are still awaited. Thus, it remains controversial whether ICIs are capable of exerting benefit by restoring the effectiveness of cellular immunity or could potentially become harmful due to the stimulation of an aberrant systemic inflammatory response.

In sharp contrast to the way that chemotherapy and radiotherapy exert their therapeutic effect, ICIs favor immunocompetence [57]. It has been suggested that the ability of ICIs to enhance the effector function of cytotoxic T-cells may explain the shorter recovery period from COVID-19 in patients with cancer receiving immunotherapy [52]. However, this may not be enough to compensate for the profound immune deregulation, including the dramatic decrease in lymphocytes seen in severe COVID-19 [58]. 

Several studies have evaluated the safety of immune checkpoint blockade in patients with cancer and concomitant COVID-19. At the early stages of the pandemic, a study of a cohort of 105 patients showed that those receiving immunotherapy had increased disease severity and mortality [59]. In another study that enrolled 423 patients with cancer, treatment with an ICI was associated with worse COVID-19 prognosis, regardless of age, cancer type, and comorbidities. However, this effect diminished when the association was adjusted for smoking, yet this conclusion is increasingly contradicted by newer studies, where no association was observed between the use of ICIs and poor COVID-19 outcomes. In a prospective cohort study of 800 patients, the severity of COVID-19 was related to age, gender, and comorbidities, but there was no evidence linking mortality to any type of therapy [60]. In a large multicenter study, cancer patients treated with ICIs had an increased risk for severe COVID-19, but immune checkpoint blockade was not a risk factor for mortality [61]. These findings received further support in a prospective study of 292 melanoma patients on ICIs, where treatment was not found to increase the risk of severe infection [62], as was the case in a large real world study of 228 patients receiving ICIs vs. 456 non-ICI treated patients, where no significant difference in COVID-19 mortality or disease severity was found [63]. These results were further corroborated by several meta-analyses investigating the incidence and mortality of COVID-19 in patients with prior exposure to an ICI [64,65]. 

It is also possible that ICIs given to patients with cancer and COVID-19 may increase the rates of immune-related adverse events (irAEs) [66,67]. There are concerns that synergy due to underlying inflammation caused by COVID-19 may increase the incidence of particular irAEs, such as pneumonitis or myocarditis [68]. This is particularly relevant for cases where combination immunotherapy is elected [69]. Furthermore, although checkpoint-inhibitor-associated pneumonitis may be hard to distinguish from COVID-19, timely initiation of appropriate therapy is essential in both cases. An observational prospective study of 293 patients concluded that the incidence of serious adverse events in SARS-CoV-2 patients receiving ICIs is significantly higher compared to non-infected patients [70].

Cancer patients are more prone to a severe COVID-19 disease course, and thus many national vaccination programs have prioritized their immunization to minimize negative outcomes. However, all patients with cancer were excluded from phase 3 COVID-19 vaccination trials, and thus vaccine immunogenicity as well as possible adverse events generated uncertainty among the patients and skepticism in their care providers [71,72,73]. Vaccine hesitancy was intensified in patients on cancer immunotherapy after initial reports of an increased number and severity of vaccine-related adverse events [74]. Subsequent studies, however, proved that the combination of COVID-19 vaccination with immunotherapy by checkpoint inhibitors is safe, with an adverse effect profile similar to healthy controls [75]. It is also worth mentioning that patients under ICIs develop, after a COVID-19 mRNA vaccination, humoral and cellular immune responses comparable to healthy controls. Considering the mounting scientific evidence, patients and treating physicians should be reassured in regard to the safety and immunogenicity of mRNA vaccination of patients receiving ICIs [76,77,78,79]. 

In conclusion, COVID-19 is associated with changes in T-cell gene expression, with alternating suppressive or activating functions seemingly depending on the disease stage. Cancer is inherently associated with increased COVID-19 severity, alongside the increased risk bestowed by the concomitant use of immunosuppressive cytotoxic treatment. On the other hand, growing evidence would suggest that treatment with immune checkpoint inhibitors does not worsen the prognosis of COVID-19. At the same time, increased vigilance and careful follow-up is needed, given the increased risk of irAEs, as well as the frequency of diagnostic dilemmas (e.g., ICI pneumonitis—COVID pneumonia) in those patients. Further research is needed to stratify patient risk for irAEs, providing data necessary for informed decision-making.

## 4. Influenza

Influenza is an acute, highly contagious, respiratory disease caused by seasonal influenza viruses that is responsible for a significant disease and economic burden [80]. In the US alone, it was estimated that during the 2019–2020 flu season, influenza caused 38 million illnesses, 40,000 hospitalizations, and 22,000 deaths. Moreover, estimates of the economic burden of influenza to the healthcare system and society in the US reach USD 11.2 billion annually [81].

The devastating effects of influenza infection are magnified in patients with cancer much more than in the general population, given the former group’s pre-existing physical vulnerability [82,83,84,85]. Furthermore, the disease disrupts the usual care and delays chemotherapy [86]. This underlying background renders vaccination vital in the preventive effort against influenza infection for cancer patients [87]. Influenza vaccination is also recommended for patients with NSCLC under ICIs, particularly after noting their higher seroconversion rates compared to patients receiving cytotoxic chemotherapy and even among the healthy population [88,89]. Additionally, in the INVIDIa study, though the incidence of an influenza-like syndrome was much higher for the vaccinated group under ICIs, their overall survival rate was also higher [90,91]. In the same study, vaccination was less efficient among the elderly but did not impact the ICI efficacy.

Data from an influenza infection in a murine model suggested that influenza infection increases the number of highly PD-1-positive innate lymphoid cells. In contrast, anti-PD-1 therapy depleted PD-1 highly positive cells and blocked papain-induced acute lung inflammation, suggesting an effective manipulation of the immune system toward both prevention and treatment of influenza infection [92]. In in vivo studies in rhesus macaque (Macaca mulatta) models, exposure to viral antigens while under PD-1 inhibition led to increased T-cell responses [93]. These results in a xenograft model supported the hypothesis that influenza vaccination increases irAEs in patients under PD-1/PD-L1 inhibition due to heightened T-cell responses [88,93]. This early concern was disproved by subsequent larger cohort studies of both the frequency and the severity of irAEs in patients under ICIs receiving influenza vaccination [89,94,95,96,97,98]. In particular, a recent meta-analysis showed that influenza vaccination appears to be a safe and reasonable intervention for cancer patients receiving ICIs, regarding both overall survival and incidence of IRAEs [99]. Very rarely, however, flu vaccine can trigger organ-specific autoimmune reactions, such us Guillain–Barré syndrome. Specifically, Yuen et al. reported a case of fatal reactivation of post-vaccination Guillain–Barré syndrome after the initiation of nivolumab [100]. This does not apply to myocarditis, a rare but serious complication that can be caused by both influenza infection and ICIs. Awadalla et al. reported that influenza vaccination was protective of myocarditis incidence in patients under ICIs. Furthermore, in the cases of ICI-induced myocarditis, the myocardial injury was limited, and the risk of major adverse cardiovascular events (MACEs) was lower among the vaccinated group compared to the non-vaccinated one [101].

## 5. HIV

HIV infection is a global public health scourge. People living with HIV (PLWH) have a notable 2.5 times higher incidence of cancer than the general population [102], and this most probably holds irrespective of smoking history [103]. Based on this evidence, NCCN guidelines strongly suggest that all patients receiving immunosuppressive therapy or chemotherapy for cancer be screened for HIV [104].

Lung cancer is the most common non-AIDS defining malignancy and the leading cause of cancer death in PLWH [102], with ICIs being increasingly used in that patient population. However, PLWH have been systematically excluded from clinical trials of ICIs, despite their increased sensitivity to chemotherapeutic toxicity, mainly because of concerns over increased irAEs and reduced efficacy due to the immunosuppressive nature of the infection [105].

This concern is not substantiated by research indicating higher PD-L1 expression and immune cell infiltration as well as more favorable immunophenotypes [106] in NSCLC tissues of PLWH. 

The available, rather small clinical data of the use of ICIs, representing mainly case reports and case series [107,108,109,110] in mostly treated aviremic HIV patients with various cancers, together with few clinical trials, including one in NSCLC [111,112] and three systematic reviews [113,114,115], have all indicated an efficacy profile at least on par with that of non-HIV patients. In particular, a recent review denoted an overall response rate of 25.9% and stable disease in 29.6% in 54 NSCLC patients [114]. A trial of durvalumab corroborated these results [111], while a nivolumab trial in 16 pretreated NSCLC patients showed a 12.5% partial response rate and stable disease in 50%. Safety regarding irAEs also seems comparable to patients without HIV. Grade 1–2 adverse events were reported in 50–70% of patients and grade 3 or higher in 6–12%, [115] with 23% of them being pneumonitis [115], a perhaps notable safety signal that needs validation. 

A single case of a fatal Kaposi sarcoma herpesvirus (KSHV)-associated polyclonal B-cell lymphoproliferative disorder after pembrolizumab use has been reported in a patient with Kaposi sarcoma and a previous KSHV-associated inflammatory cytokine syndrome [112]. However, we should be cognizant of the relative lack of evidence of safety or efficacy in severely immunocompromised (CD4+ T-cell count <100) patients.

In infected HIV patients, the virus challenges the immune system in unique ways. Viral reservoirs (mainly CD4+ memory and follicular helper T-cells in lymph nodes) [116] contain the latent replication-competent provirus that is shielded from immune recognition and antiretroviral therapy (ART). HIV-specific cytotoxic CD8+ T-cells exist in a functionally exhausted state, with reduced replicative capacity and increased sensitivity to apoptosis [117]. This state is thought to be maintained due to a persistent antigen load, chronic inflammation (in which protein products of replication-incompetent proviruses might play an important role [118]), and loss of CD4+ help [119]. Consequently, the residual viral burden of patients under treatment cannot be fully eliminated and the prospect of viral cure is hampered.

**Figure 2:**
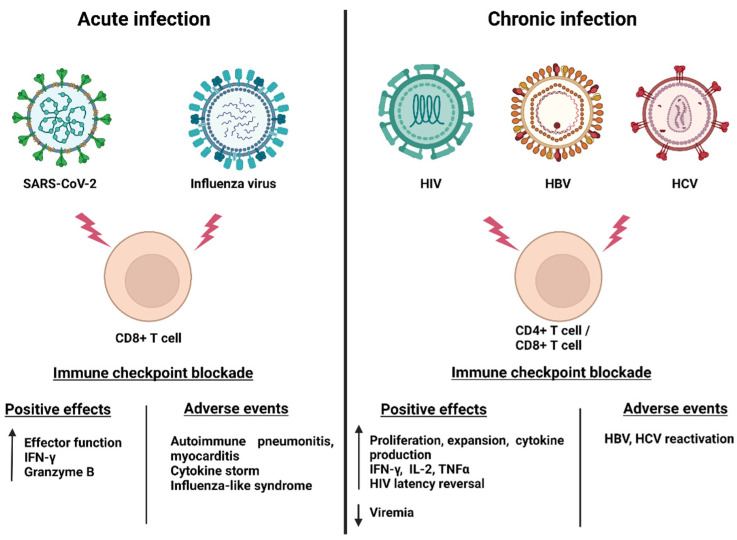
Effects of ICB therapy in chronic and viral infections. ICB: immune checkpoint blockade.

Immune checkpoint receptors seem to contribute to the viral evasion of the immune system. HIV-specific CD8+ Τ-cells that show an exhausted phenotype express PD-1 [117,120,121] in an antigen-dependent fashion [117], and ART only partially reduces its expression [122]. Additionally, CD4+ T-cells that express PD-1 (and other immune checkpoint molecules such as LAG-3 and TIGIT) are enriched for HIV DNA [123,124,125], making immune checkpoint receptors valuable markers of the latent reservoir. They also seem to be involved in the reservoir persistence [126]. In that context, use of immune checkpoint blockade could potentially reinvigorate T-cell function and reverse HIV latency, two aspects of the “shock-and-kill” approach of a functional HIV cure [127,128]. Figure 2 summarizes the effects of ICIs in HIV and other chronic or acute viral infections.

In preclinical studies, use of an anti-PD-L1 antibody led to augmented proliferation of CD4+ and CD8+ HIV-specific T-cells and functional amplification of CD8+ T-cells [117,120]; anti-PD-1 and PD-L1 antibodies led to an increase in survival of CD8+ HIV-specific T-cells [129]. Ex vivo blockade of PD-L1 and PD-1 did not bring about consistent latency reversal in CD4+ Τ cells of treated patients [130], but it did so in combination with a latency reversal agent [126].

In animal studies, PD-1 blockade led to enhanced antiviral CD8+ T-cell function and a faster reduction in viral loads, reduction in viral reservoir, and delayed viral rebound when used in ART-treated SIV-infected rhesus macaques (RMs) [131]. It also reduced the viral load and prolonged survival in untreated RMs [132] and led to significant viral load reduction and CD8+ T-cell proliferation in humanized mice [133]. In contrast, dual ICR blockade (PD-1/CTLA-4) in aviremic ART-treated RMs led to latency reversal and partial reduction in the reservoir, as evidenced by plasma viremia and reduction in integrated HIV DNA in CD4+ T-cells, but it did not slow down a viral rebound after ART interruption [134].

The mixed results of IC blockade in preclinical studies are mirrored in the data from the few available virologic studies in patients, as is suggested from the aforementioned clinical research. In these studies [111,112,113,114,115], in which a large majority of patients had undetectable viral loads under ART, CD4 counts remained essentially stable, and plasma viral loads showed minimal non-sustained surge or no change at all, establishing a template of virologic safety. In a recent systematic review [114], just 15.8% of 76 patients with undetectable HIV RNA plasma viral load showed an increase to a level less than 400 copies/mL during ICI treatment.

The effect on the infection itself has been studied in a trial of an anti-PD-L1 agent, BMS-936559, in HIV patients without malignancies receiving ART. Two of the six patients had a slight increase (0.09%) in IFNγ expression in Gag-specific CD8+ T-cells, but plasma viral load and cell-associated RNA/DNA were unchanged, indicating a lack of effect on the viral reservoir [135]. 

In a single case report, an HIV patient treated with nivolumab for lung cancer had a sustained and significant (1 log10) decrease in cell-associated DNA within three months after initiation of treatment, together with a parallel increase in the number of HIV-specific T-cells and a transient increase in plasma viral load, all of which would suggest a decrease in the viral reservoir [136]. In contrast, another nivolumab treated lung cancer patient had a slight increase in HIV-specific CD8+ T-cells with no apparent effect on the viral reservoir [137].

Three other virally suppressed patients with squamous carcinomas showed no change in HIV DNA and minimal HIV/Gag-specific T-cell responses after multiple doses of anti-PD-1 therapy [138]. Additionally, virologic analysis of 29 participants of an aforementioned phase I pembrolizumab study [112] also revealed only transient effects on unspliced cell-associated RNA and viral DNA (increased on D7 but not on D21, decreased on D1 but not D7, respectively). Lastly, recently published virologic data of a phase I clinical trial of nivolumab alone or in combination with the anti-CTLA4 agent ipilimumab in aviremic PLWH with various cancers [139] indicated no latency reversal or reservoir reduction potential of nivolumab alone, but the combination resulted in a modest 1.44-fold increase in cell RNA 24 h after the first dose, indicative of latency reversal (six of seven patients) and a 97% and 64% longitudinal decrease in cells containing replication-competent HIV in two patients with available measurements.

The sparse data presented above are too limited for firm conclusions but imply that anti-PD-1/PD-L1 inhibition in patients with HIV and cancer is safe and effective, though with varied effects on virological outcomes (mainly no effect or isolated HIV-specific CD8+ T-cell increases) and is unlikely to be the sole part of an HIV cure strategy [114]. The diversity and the lack of standardization of some methods used for quantification of the HIV reservoir are also notable factors that contribute to the heterogeneity of the results.

Several ongoing trials of ICIs in PLWH with or without malignancies will probably further elucidate their role in the treatment of HIV [140].

## 6. Hepatitis B and Hepatitis C 

There are approximately 290 million people around the world living with hepatitis B virus (HBV) infection and 80 million with hepatitis C virus (HCV) infection [141]. The prevalence of viral hepatitis is higher among patients with malignancies of the liver, gastrointestinal tract, head and neck, and lung and prostate, however, with the specific frequencies dependent upon the type of viral hepatitis. Chronic hepatitis C infection is related to hepatocellular carcinoma (HCC), while eradication of the hepatotropic virus with direct acting antivirals improves survival when HCC patients are infected by other viruses such as COVID-19 [142]. Only 8% of concomitant malignancy and viral hepatitis cases had their cancer treatment modified because of the viral status of the patients [143]. Previous clinical trials of immune checkpoint inhibitors excluded active HBV/HCV-infected patients due to the potential of reactivation and subsequent immune-mediated hepatic failure, a life-threatening condition [144,145].

The T-cellular adaptive immune response plays an important role in the development of HBV and HCV infections. CD4+ and CD8+ T-cell mediated responses are important for viral clearance in both infections [146]. CD4+ T-cell exhaustion leads to viral persistence in both [147,148], as they indirectly contribute to viral clearance of HBV and HCV by maintaining and enhancing the antiviral activity of CD8+ cells. CD4+ depletion leads to persistent viral infection [149,150]. CD8+ T-cells are crucial for the clearance of HBV and HCV, and CD8+ T-cell exhaustion is associated with higher viral loads in both infections [151]. CD8+ cells in chronic HBV and HCV infection gradually become dysfunctional, compromising the adaptive immune system’s ability to control the infection [152,153]. As observed in a chimpanzee xenograft model, the depletion of CD8+ cells led to chronic infection, indicating their crucial role in the pathogenesis and progression to chronicity [154,155]. On the other hand, this corroborates the connection between the functional state of the cells and the clearance of the virus [154,156].

CD4+ and CD8+ T-cells in patients with chronic HBV infection express PD-1, CTLA-4, and TIM3, whereas circulating HCV-specific CD8+ T-cells display upregulated expression of PD-1 [157]. PD-1 expression is elevated in the hepatocytes of patients with chronic HBV and HCV infection, making a suggested seminal contribution to the dysregulation of CD8+ cells [158,159]. Thus, PD-1/PD-L1 inhibitors have a potential place in the immunotherapy of chronic hepatitis. Blockade of the PD-1/PD-L1 pathway led to the restoration of interferon-γ production by CD8+ cells and the restoration of functional CD8+ cells in mice and in vitro [160,161].

There are reports of reduced viral load in patients with HBV and HCV infections who were treated with anti-PD-1 monoclonal antibodies for metastatic melanoma or non-small-cell lung cancer [162]. There are several reports of HCV reactivation after anti-PD-1 treatment, but there is no systematic analysis to confirm the risk for HCV patients under ICIs [163]. A recent systematic analysis showed that HBV reactivation occurs in 5.3% of HbsAg-positive patients who were undergoing anti-PD-1/PD-L1 therapy and did not receive antiviral therapy [164]. The mechanism is not entirely clear. Blockade of the PD-1/PD-L1 axis may lead to the restoration of CD8+ cell function [165] but also exacerbation of liver damage via cell mediated cytotoxicity. The destruction of hepatocytes can lead to the release of previously latent virus into the circulation [166,167]. According to the ASCO guidelines, all cancer patients should be tested for hepatitis B surface antigen (HBsAg), hepatitis B core antibody (anti-HBc) total immunoglobulin (Ig) or IgG, and antibody to hepatitis B surface antigens (anti-HBs) before or at the beginning of systemic anticancer therapy [168].

## 7. Conclusions

The fact that both cancer and viral infections exploit similar, if not the same, immune checkpoint molecules to escape the host immune response highlights the need to study the impact of immune checkpoint blockade on viral infections. Immunotherapy has been under investigation as a potential treatment for both viral and bacterial infections for a considerable amount of time. However, the results of many clinical trials were inconclusive, and thus immunotherapy has not been established as a treatment option in the therapeutic algorithm of any infection. This has recently been changed, since three biological agents have received approval as an effective and safe COVID-19 treatment [169,170,171,172,173]. Moreover, the combination of approved or experimental vaccines with ICIs has proven to be a promising approach to improve vaccine immunogenicity and efficacy [174]. The development of companion diagnostics to simultaneously detect immune checkpoint expression and viral load is essential and would potentially require the resurfacing of older, well-established technologies such as DNA/RNA microarrays. More importantly, the process through which immune checkpoint blockade completely changed the way we approach cancer could be the key to decipher the potential role of immunotherapy in the therapeutic algorithm of viral infections.

## Figures and Tables

**Table 1:** Anti-PD-1/PD-L1 agents used in viral infections clinical trials.

Type of Infection	Virus	ICI Agent	Target	Trial Identifier	Number of Patients	Primary Endpoint
Acute	SARS-CoV-2	Nivolumab	PD-1	(NCT04356508)	15	Viral clearance kinetics
		Pembrolizumab	PD-1	(NCT04335305)	12	Efficacy and safety of tocilizumab combined with pembrolizumab in patients with COVID-19 pneumonia
		Camrelizumab	PD-1	(NCT04268537)	120	Lung injury score
	Influenza virus	Pembrolizumab, nivolumab	PD-1	(NCT03590808)	136	Seroprotection rate after influenza vaccination
Chronic	HIV	Pembrolizumab	PD-1	(NCT02595866)	32	Measurement of unspliced HIV RNA, RNA/DNA ratio before and after treatment
		Nivolumab/nivolumab + ipilimumab	PD-1	(NCT02408861)	33/7	Measurement of unspliced HIV RNA and DNA during treatment, replication competent virus before and after treatment
		Durvalumab	PD-L1	(NCT03094286)	20	Feasibility of durvalumab monotherapy in solid tumors for HIV-1-infected patients
		BMS-936559	PD-L1	(NCT02028403)	8	− Occurrence of adverse events − Frequency of HIV-1 Gag-specific CD8 T-cells at baseline and after treatment − HIV-1 at baseline and after treatment
		Cemiplimab	PD-1	(NCT03787095)	5	− Occurrence of adverse events − Early termination due to adverse events
	HBV	Durvalumab	PD-L1	(NCT03899428)	30	Reduction in serum HBsAg titers
	HCV	MDX1106-02	PD-1	(NCT00703469)	54	Safety, tolerability, and immunogenicity
